# Night Shift: Expansion of Temporal Niche Use Following Reductions in Predator Density

**DOI:** 10.1371/journal.pone.0038871

**Published:** 2012-06-13

**Authors:** Douglas J. McCauley, Eva Hoffmann, Hillary S. Young, Fiorenza Micheli

**Affiliations:** 1 Hopkins Marine Station, Stanford University, Pacific Grove, California, United States of America; 2 Center for the Environment, Harvard University, Cambridge, Massachusetts, United States of America; Texas A&M University, United States of America

## Abstract

Predation shapes many fundamental aspects of ecology. Uncertainty remains, however, about whether predators can influence patterns of temporal niche construction at ecologically relevant timescales. Partitioning of time is an important mechanism by which prey avoid interactions with predators. However, the traits that control a prey organism's capacity to operate during a particular portion of the diel cycle are diverse and complex. Thus, diel prey niches are often assumed to be relatively unlikely to respond to changes in predation risk at short timescales. Here we present evidence to the contrary. We report results that suggest that the anthropogenic depletion of daytime active predators (species that are either diurnal or cathemeral) in a coral reef ecosystem is associated with rapid temporal niche expansions in a multi-species assemblage of nocturnal prey fishes. Diurnal comparisons of nocturnal prey fish abundance in predator rich and predator depleted reefs at two atolls revealed that nocturnal fish were approximately six (biomass) and eight (density) times more common during the day on predator depleted reefs. Amongst these, the prey species that likely were the most specialized for nocturnal living, and thus the most vulnerable to predation (i.e. those with greatest eye size to body length ratio), showed the strongest diurnal increases at sites where daytime active predators were rare. While we were unable to determine whether these observed increases in diurnal abundance by nocturnal prey were the result of a numerical or behavioral response, either effect could be ecologically significant. These results raise the possibility that predation may play an important role in regulating the partitioning of time by prey and that anthropogenic depletions of predators may be capable of causing rapid changes to key properties of temporal community architecture.

## Introduction

Predation is believed to have played an important role in shaping the long-term evolution of diel cycles and temporal niche partitioning in animals. For example, a major factor that contributed to the nocturnal disposition of multiple species appears to have been the capacity to avoid daytime active predators [1], [2]. Presumably many patterns in temporal niche separation that arose from historical predator/prey interactions continue to be enforced today by contemporary predation risk.

Generally there is much interest amongst ecologists in understanding how key properties of community organization and function, such as diel partitioning, change when the drivers that brought them about are relaxed. In the case of predation there is considerable field and experimental evidence demonstrating that a variety of basic life history traits (e.g. growth and reproduction) can evolve rapidly when predation pressure is reduced [3]–[5]. There has, however, been limited research on whether diel behavior and patterns of temporal niche partitioning can also undergo rapid change following modulation of predation risk [6], [7]. The dynamics of the relationship between diel behavior and predation deserve more attention given that predators in myriad ecosystems are being rapidly depleted by humans [8], [9].

Here we engage these issues by considering whether nocturnal animals are capable of behaviorally or numerically responding to reductions in predation risk from daytime active predators. To do this we compared the relative abundance of nocturnal prey fish communities observed during the day at a coral reef ecosystem where large daytime active predators were abundant to those on physically similar reefs where these predators have been fished to low levels in the last three decades. Our results suggest that predators may indeed play an important role in enforcing the boundaries of nocturnal prey niches. Such observations challenge us to more broadly consider the controls that predators may have upon fundamental aspects of niche construction and inform our understanding of the full extent to which anthropogenic change is capable of impacting animal ecology.

## Methods

This work was carried out under permission from USFWS SUP # 12533-06032 and from the Republic of Kiribati Environment and Conservation Division. This research was conducted at Palmyra (USA; 5° 52′ N, 162° 04′ W) and Tabuaeran (Kiribati; (3° 51′ N, 159° 19′ W) Atolls. Palmyra is a US National Wildlife Refuge and prohibits the take of all reef fish. The reefs of Palmyra are among the least disturbed in the world and host especially high densities of large predatory fish [10], [11], [12]. Tabuaeran, located 350 km southeast of Palmyra, is a lightly populated atoll that hosts approximately 2,500 persons (Secretariat of the Pacific Community, Kiribati 2005 Census Volume 2: Analytical Report). Densities of many large fish, particularly sharks and other top predators, have been much reduced at Tabuaeran by fishing [10]. The bulk of these reductions appear to have taken place in the last several decades and were associated with the arrival of a mass influx of new residents to Tabuaeran during government settlement programs in the 1980's and 90's [13]. These two nearby atolls are otherwise physically, chemically, and oceanographically similar to one another [13].

We used SCUBA belt transect surveys to inventory fish assemblages during full light conditions (1000 – 1600 h) on the forereefs (ocean-side of reef crest) of both Palmyra and Tabuaeran. A complete fish survey consisted of four belt transects, the dimensions of which were matched to size (total length; TL) of individual fish being inventoried: ≥50 cm TL fish −50×8 m transect; 30–49 cm TL fish −50×4 m transect; 15–29 cm TL fish −50×4 m transect; <15 cm TL fish −25×2 m transect [14]. All transects were run along a 10–12 m depth isobath. In each transect two divers identified, counted, and estimated the total length of individual fish. Nine forereef sites were surveyed seven times each at Palmyra (along north and south shores) and five forereef sites were surveyed four times each at Tabuaeran (along west and south shores). Sites were evenly spaced ∼ 2 km apart from one another at random locations. Fish surveys were conducted at Palmyra between June – Aug 2006 and at Fanning between Mar –April 2007; replicate surveys were evenly temporally dispersed across these periods. The same two divers conducted all surveys at both atolls. Fish biomass was estimated from survey data using length-weight conversion constants obtained from FishBase (Froese and Pauly 2009) or other published literature. Each species of fish observed in these surveys was classed as either: 1) “nocturnal”– >75% of its feeding and activity takes place at night (dusk to dawn); 2) “diurnal”–>75% of its feeding and activity takes place during the day (dawn to dusk); or 3) “cathemeral”–all other fish, i.e. species active during the day and night. These assignments were made using data from FishBase, extensive reviews of published literature, and surveys of expert opinion (Supporting dataset S1).

Fish within each of these three categories differ in their degree of conformity and specialization to the specified diel modes. In the case of nocturnal fishes, the ratio of eye diameter to fish standard length (SL) serves as a convenient proxy for picking out gradients in adherence to nocturnal living [15]–[17]. Animals that have larger eyes relative to their body length are generally thought to have better visual acuity in dim light situations and be more strictly nocturnal [18]. Many of the physiological adaptations that permit these especially nocturnal fish to function in low light environments (e.g. increased rod density, reduced cone density, specialized spectral sensitivity, changes in focal length [16], [19]) may make their vision less well suited for high light environments. Thus, the differential investment in nocturnal vision by fish with large eye: SL ratios likely make such species especially vulnerable to predation during the day (as seen in reverse for refuging diurnal species [20]) and more responsive to the removal of large daytime active predators. To examine these hypotheses, we collected data on the ratios of eye diameter (widest part of the eyeball along anterior-posterior axis) to SL for all of the fish genera encountered at Palmyra and Tabuaeran from museum specimens (California Academy of Sciences) and from published values [17]. Species for which values were not reported in the literature were taken from two individuals from each of two species per genus.

Differences in the density and biomass of diurnal, cathemeral, and nocturnal fish species were compared between Palmyra and Tabuaeran using effect size measurements (Cohen's D with pooled standard deviation; Supporting Text S1). We also measured differences in fish abundance between atolls by comparing the percentage of surveys in which each species was sighted (i.e. >0 individuals observed at any point during the survey dive). To gauge how fishing by the residents of Tabuaeran may have impacted the abundance of large predators capable of feeding on nocturnal prey, we compared the biomass of all “large predators” (piscivorous fish ≥10 kg; size at which predator gape most reasonably permits the capture of all size classes of nocturnal fishes) observed during fish surveys at both atolls. All of these large predators were daytime active (i.e. either diurnal or cathemeral) and the majority were cathemeral (Supporting dataset S1). To search for direct associations between nocturnal fish abundance responses and predator abundance, we regressed the density effect size of all fish species against their eye diameter to SL ratio. Large predators were excluded both from these regressions and effect size analyses.

The significance of these effect size comparisons was evaluated using Kruskal-Wallis and Wilcoxon rank sum tests – as data could not be transformed to meet parametric assumptions. Post-hoc Holm's sequential Bonferroni corrections [21] were applied to interpret significance levels (3 groups: diurnal (D) vs cathemeral (C) vs nocturnal (N)). All statistics were computed in Program R (R Development Core Team (2010), http://www.R-project.org).

## Results

**Figure 1:**
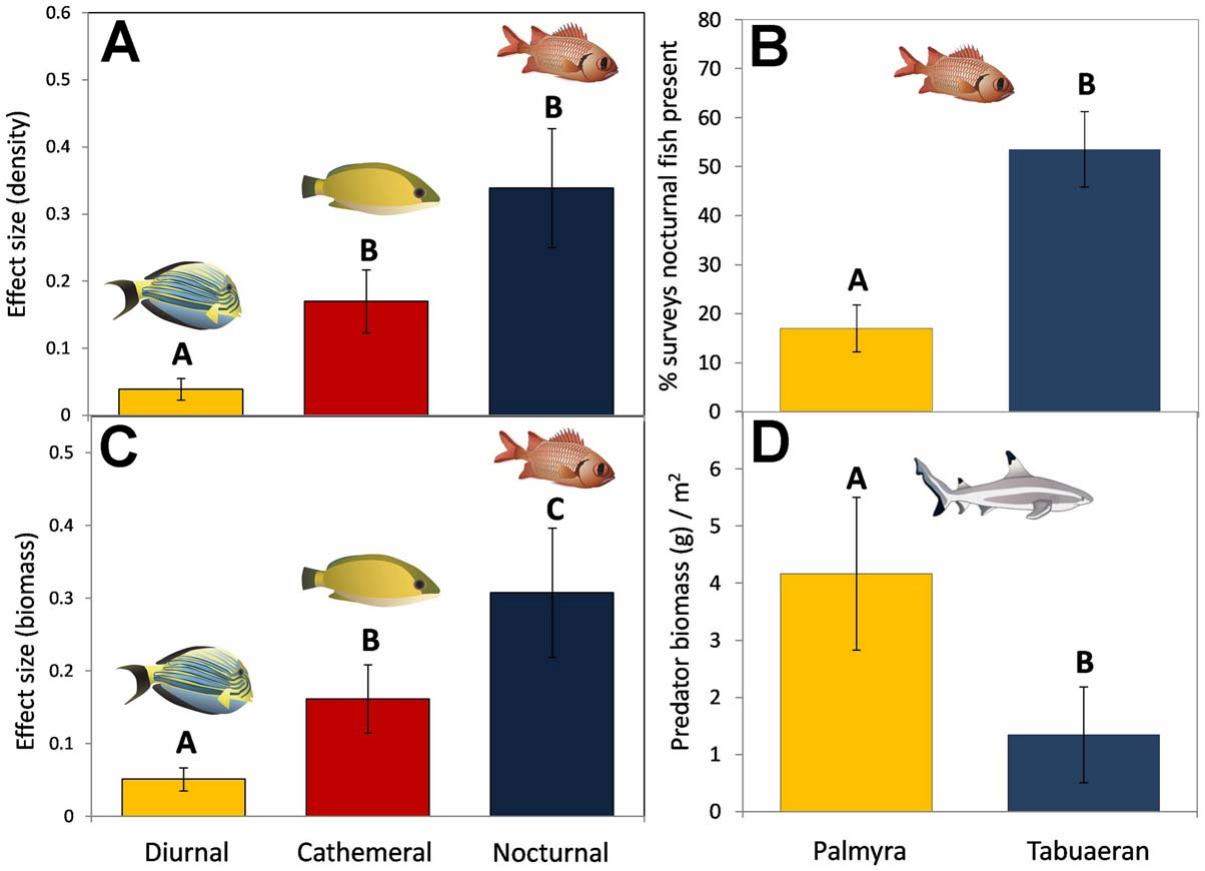
Effect size plots representing differences in the density (A) and biomass (C) of nocturnal, diurnal, and cathemeral prey fish on reefs at predator-depleted Tabuaeran Atoll relative to predator-heavy Palmyra Atoll. Positive values represent increases at Tabuaeran. Percentage of surveys (B) in which nocturnal prey fish were sighted at Palmyra and Tabuaeran. Comparisons of the biomass of all large (≥10 kg) diurnal and cathemeral piscivorous predators (D) at both atolls. All values are mean, ±1 SE. Surveys marked with the same letters in each species grouping are not significantly different (after post-hoc correction).

Of the 185 species that were shared between Palmyra and Tabuaeran we classified 141 as diurnal species, 26 as cathemeral species, and 18 as nocturnal species (Supporting dataset 1). Nocturnal fish included representative species from 6 families: Holocentridae, Priacanthidae, Pempheridae, Lethrinidae, Mullidae, and Serranidae. No nocturnal fish in this assemblage achieved a SL >50 cm (mean size  = 22.4 cm SL, ±5.19 SD), generally qualifying all as potential prey for large piscivorous reef fish. Data from our fish surveys indicated that large daytime active predators were abundant on the reefs of Palmyra, but were considerably depleted at Tabuaeran (Fig. 1d; W = 108, *P* = 0.04). This finding parallels observations made by other researchers that have reported pronounced anthropogenic depletions of large piscivores at Tabuaeran [10], [11].

Analysis of the effect size patterns of diurnal, cathemeral, and nocturnal fish species indicated that nocturnal fish showed by far the strongest increases in both density and biomass during the day at Tabuaeran relative to Palmyra (Fig 1a and 1c). These increases exhibited by nocturnal fish were significantly greater than the abundance increases shown by diurnal species (density: N vs D, W = 2191, *P*<0.0001; biomass: N vs D, W = 2175, *P*<0.0001) but only significantly different (post-correction) from cathemeral species in the case of biomass (density: N vs C, W = 253, *P* = 0.035; biomass: N vs C, W = 258, *P* = 0.022). Abundance increases in cathemeral species between Tabuaeran and Palmyra were intermediate to nocturnal and diurnal species, and always significantly higher than the diurnal increase (density: C vs D, W = 1023, *P* = 0.043; biomass: C vs D, W = 980, *P* = 0.025). Presence/absence data summarized from these fish surveys conveyed a similar conclusion. Nocturnal fish were sighted in considerably more surveys at Tabuaeran than Palmyra (Fig 1b; W = 49, *P*<0.001). Cathemeral species were also sighted at higher frequencies at Tabuaeran (W = 115, *P* = 0.02) but diurnal species were not different between atolls (W = 10196 *P* = 0.87).

**Figure 2:**
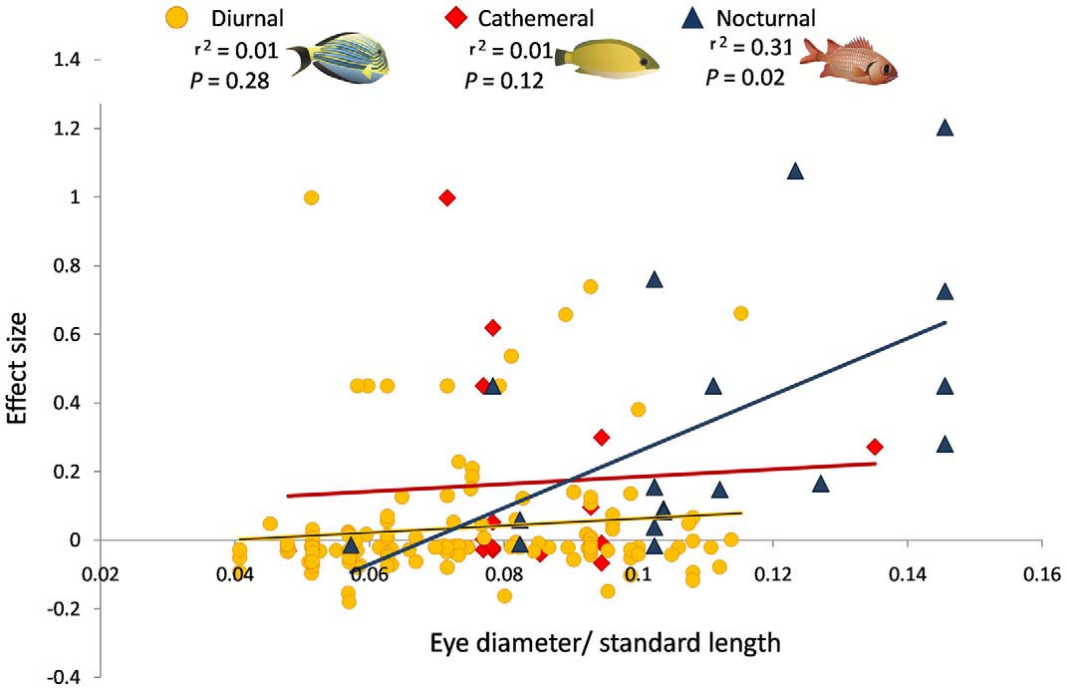
Effect sizes of reef fish density regressed against the ratio of fish eye diameter to standard length (SL). Large effect size values represent strong increases in density at predator impoverished Tabuaeran Atoll. Nocturnal, diurnal and cathemeral are segregated in the plot. Fish with large eye diameter: SL are thought to be increasingly well adapted for functioning at night. The most dark adapted fish showed the strongest responses to predator depletions. There was no significant relationship between effect size response and eye diameter: SL for diurnal or cathemeral fishes.

We observed a significant positive relationship between eye diameter: SL ratios and the density effect size response of nocturnal reef fish species. This observed relationship indicates that the most dark adapted species, which are expected to be more vulnerable to daytime active predators, showed the strongest increases in abundance at Tabuaeran (Fig 2). No such relationships were found when comparisons were made only with diurnal or cathemeral species (Fig 2). When the same regression was run for all fish without regard to diel class, a significant but less positive relationship was observed (*R^2 = ^*0.12, *P*<0.0001).

## Discussion

**Figure 3:**
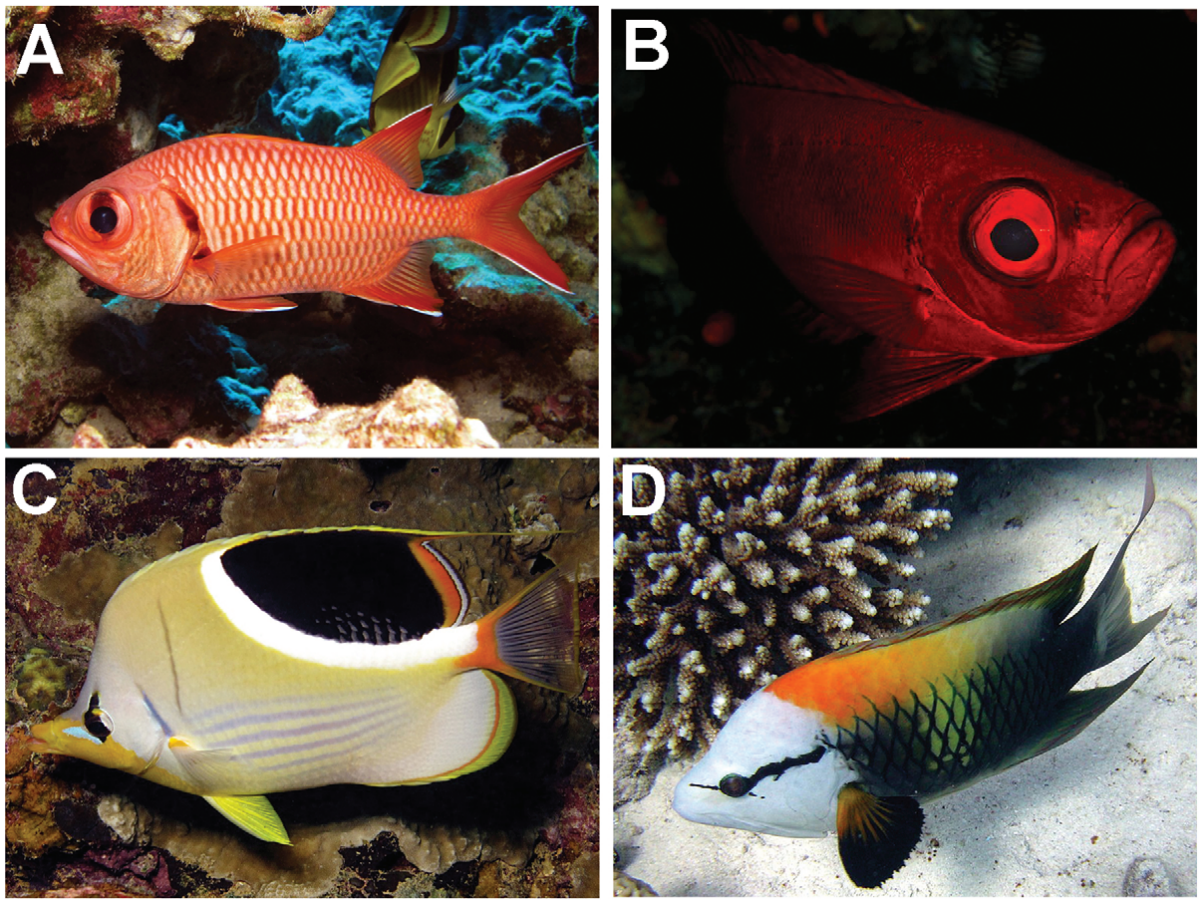
Examples of two species of nocturnal reef fish surveyed in this study: *Myripristis berndti* (A) and *Priacanthus hamrur* (B). The large eye to body length ratios of these two species (*M. berndti*: 0.15; *P. hamrur*: 0.11) give evidence of their nocturnal lifestyle. Like many nocturnal fish, *M. berndti* and *P. hamrur* both dramatically increased in abundance at Tabuaeran where large daytime active predators were less abundant. Example diurnal fish *Chlorurus sordidus* (C) and *Epibulus insidiator* (D) exhibit the smaller eye to body length ratios (*C. sordidus*: 0.05; *E. insidiator*: 0.05) that are more characteristic of diurnal reef fish. These species were among the diurnal prey fish taxa whose abundance showed negative or weak responses to predator depletion.

Our observations suggest that reductions in predator density in coral reef ecosystems can facilitate the temporal niche expansion of prey species. While these conclusions are drawn from the study of only two systems, the resultant observations are quite compelling. At our Tabuaeran Atoll study sites where fishers have depleted large diurnal and cathemeral predatory fish, we observed strong increases in the daytime density, biomass, and sighting frequencies of nocturnal prey fish compared to our sites at Palmyra Atoll where daytime predators remained naturally abundant (Fig. 1 and 3). Diurnal or cathemeral prey fish species also appeared to benefit and increase in abundance (density and biomass) at Tabuaeran where predators were fewer – but these increases were not nearly as dramatic as the increases observed for nocturnal prey guilds. We suggest that the most parsimonious explanation for this pronounced response by nocturnal prey fish is that the removal of large daytime active predators on the fished reefs of Tabuaeran allowed nocturnal prey fish in this system to increase during portions of the day when they are normally highly vulnerable to such predators.

Without data on the abundance of nocturnal prey fish at Tabuaeran during the periods prior to the depletion of its large predators, or the capacity to engineer exclosure manipulations large enough to meaningfully capture large scale interactions between reef predators and nocturnal prey fish, we cannot directly identify the mechanisms that have caused the stark differences in nocturnal prey fish abundance on Tabuaeran's predator depauperate reefs. However, a predatory release explanation for these shifts is well supported by the relationships observed between degree of specialization for nocturnal living by prey fish (as proxied by eye diameter: SL values) and the effect sizes of their abundance responses to predator reductions. If risk of predation from daytime predators is indeed playing an important role in constricting the diel niches of nocturnal fishes at these sites, we would expect the nocturnal species that are most vulnerable to daytime predators (i.e. those that are particularly specialized for nocturnal living) to show the strongest responses when these predators are reduced in number. This was indeed what was observed: nocturnal prey fish species that were highly adapted to functioning at night and presumably most at risk to predation during the daytime (i.e. fish with large eye diameter: SL values) showed the strongest positive responses to the predator reductions at Tabuaeran (Fig 2). Other alternative explanations for these observed changes in nocturnal fish abundance are less well supported. The species richness of reef fish communities at our predator rich and predator depleted reefs was largely the same, obviating concerns that naturally varying differences in fish diversity generated this pattern. Furthermore, the oceanographic similarity of these atolls [13] provides little reason to believe that the quantity or timing of delivery of nocturnal fish prey (e.g. plankton) would systematically differ in such a way as to affect the temporality of their foraging. It is possible that resource competition within temporal feeding guilds may have played some role in creating the patterns we observed (e.g. nocturnal prey become more abundant in the absence of large predators and must begin feeding during the day to cope with density mediated increases in competition from other nocturnal fish). However, changes in competitive regimes seem unlikely to be the primary driver for the patterns we observed in daytime abundance of nocturnal fish at Tabuaeran, given the specifically pronounced responses of fish species with large eye diameter: SL ratios.

There is no reason to believe that nocturnal fishes are the only taxa whose temporal niche space would be affected by the removal of reef predators at Tabuaeran. Observations made in this same archipelago have demonstrated that diurnal prey fish undergo dramatic shifts in behavior (i.e. excursion distance) in the environments where predation risk is minimized [22], [23]. Further investigation (e.g. night surveys of diurnal fish) will be required to determine whether other diel fish guilds or other reef taxa have undergone shifts similar to those observed in nocturnal fish assemblages.

It is not possible to determine conclusively whether the changes we observed in the nocturnal fish communities of Tabuaeran were principally the result of alterations in their abundance or their behavior. Nocturnal fish cannot be counted effectively using non-destructive methods while in diurnal refuges [24] and safety regulations prevent nighttime diving at Palmyra. These limitations impeded our ability to collect data on the “actual” abundances of nocturnal fishes at Palmyra and Tabuaeran that could be used to identify the mechanistic origins of this change. However, either a numerical or behavioral response by these nocturnal assemblages would be biologically interesting.

Determining the ecological consequences of these diurnal increases in the abundance of nocturnal predators will require gathering more data on the foraging efficiency of these nighttime feeders. The fish species with large eye: SL ratios, which showed the strongest increases in abundance on predator depleted reefs, are also likely to be the poorest daytime foragers given the physiology of their vision. These taxa may be weak competitors with more light-adapted diurnal species feeding on similar prey. Future research will help resolve how the dynamics of both competition and resource availability in this reef system may be altered by these increases in the abundance of nocturnal consumers.

Rapid changes in patterns of diel partitioning following disruptions in predator regimes have been observed in mammals [25], [26], invertebrates [27]–[30], and freshwater fishes [6], [31], [32]. Temporal niche shifts have not, however, been previously reported for coral reef fish, nor have they ever been observed to advance so synchronously across multiple taxa as they did in this system. In fact, with the exception of the few above examples, rapid changes in diel behavior following predator manipulation are very rare [33] – a somewhat curious observation given the frequency and intensity of modern anthropogenic alterations to predation risk regimes [8], [34]. Why then do the effects that we observed at Tabuaeran appear to be so rare elsewhere? There are at least four non-mutually exclusive explanations for why we do not see more examples of temporal niche shifts in a world where large predators have been removed from numerous ecosystems. First, the traits required to successfully undergo shifts in diel activity rhythms may, in some taxa, be deeply entrenched phylogenetically and resistant to rapid evolution [35]. Second, these types of shifts may be widespread, but taking place at such slow rates that they have escaped the notice of researchers. Third, alterations in the diel behavior of prey may not be recognizable because of the global rarity of less-disturbed, predator rich systems that are required to recognize such change. Lastly, it is quite possible that in many instances humans functionally replace the non-human predators they extirpate [36] and continue to enforce existing patterns of diel partitioning by hunting the prey of these depleted predators themselves.

The possibility that changes to predation regimes can alter well-established patterns of temporal partitioning raises many interesting questions. Do prey and competitors respond to these rapid shifts in temporal niche space, causing evolutionarily significant temporal cascades in ecosystems [37]? Do changes in physiology track these observed changes in temporal partitioning by predators? Can these contemporary changes in diel activity help explain historical patterns of temporal niche evolution (e.g. the rapid expansions in reef fish nocturnality in the Eocene [38])? Answering these types of questions will shed more light on the ecological and evolutionary significance of these first observed steps out of the darkness by nocturnal prey.

Future research on the effects of predators on the temporal ecology of prey will be necessary to examine the geographic and taxonomic ubiquity of the trends we report herein. Developing this line of research is critically important given the rapidity and severity by which humans are depleting predator populations in both marine and terrestrial environments [8], [9], [39]. These compelling first observations from our study sites at Palmyra and Tabuaeran suggest that anthropogenic change may be affecting elements of ecology as foundational as diel activity patterns and thus challenge us to expand the scope at which we consider how humans may be altering communities.

## Supporting Information

Dataset S1 **Temporal niche classifications of the fishes at predator rich Palmyra and predator depauperate Tabuaeran Atolls.** Comparisons of the density and biomass of these fish species, as conducted using effect size measurements, are also reported.
(DOC)

Text S1 **Mechanics of Cohen's D effect size measurements used to compare the density and biomass of fish species on the reefs of Palmyra and Tabuaeran.**
(DOCX)
