# Supplementary material for: Night Shift: Expansion of Temporal Niche Use Following Reductions in Predator Density
Source: PLoS One. 2012 Jun 13;7(6):e38871. doi: 10.1371/journal.pone.0038871 (PMC3374761; doi:10.1371/journal.pone.0038871)
Supplement: Dataset S1 — Temporal niche classifications of the fishes at predator rich Palmyra and predator depauperate Tabuaeran Atolls. Comparisons of the density and biomass of these fish species, as conducted using effect size measurements, are also reported. (DOC) [file pone.0038871.s001.doc]

SUPPORTING INFORMATION

**Supporting Dataset S1.** Temporal niche classifications of the fishes at predator rich Palmyra and predator depauperate Tabuaeran Atolls. Diel classifications: N = nocturnal; D = diurnal; C = cathemeral. Diel assignments were based on data from FishBase (Froese and Pauly 2011), personal observation, expert opinion, and the following literature sources: Hiatt and Strasburg 1960, Hobson 1974, Harmelin-Vivien and Bouchon 1976, Steene 1978, Compagno 1984, Shpigel and Fishelson 1989, Randall et al. 1990, Heemstra and Randall 1993, Lieske and Myers 1994, Holland et al. 1996, Myers 1999, Kuiter and Tonozuka 2001, Marnane and Bellwood 2002, Randall 2005, and Gillibrand et al. 2007. Effect sizes for density and biomass of all fishes are also displayed and were calculated with Cohen’s D effect size formula (Supporting Text S1) such that positive effect size values represent increases in fish density or biomass at Tabuaeran. * = fish predators ≥ 10 kg. Scientific names follow the taxonomy of Randall 2005 and FishBase.

| **Species** | **Diel**  **Classification** | **Density Effect size** | **Biomass Effect size** |
| --- | --- | --- | --- |
| *Abudefduf sordidus* | D | 0.0176 | -0.0193 |
| *Acanthurus achilles* | C | -0.0280 | -0.0223 |
| *Acanthurus blochii* | D | -0.0392 | -0.0439 |
| *Acanthurus guttatus* | D | 0.4497 | 0.4497 |
| *Acanthurus leucocheilus* | D | -0.0461 | -0.0398 |
| *Acanthurus lineatus* | D | 0.0696 | -0.0004 |
| *Acanthurus maculiceps* | D | -0.0479 | -0.0426 |
| *Acanthurus nigricans* | D | 0.0073 | 0.0521 |
| *Acanthurus nigricauda* | D | 0.0552 | 0.0788 |
| *Acanthurus olivaceus* | D | -0.0463 | -0.0338 |
| *Acanthurus thompsoni* | D | 0.0140 | 0.0432 |
| *Acanthurus triostegus* | D | 0.0000 | 0.0000 |
| *Acanthurus xanthopterus* | D | -0.0761 | -0.0712 |
| *Aluterus scriptus* | D | 0.0000 | 0.0000 |
| *Amanses scopas* | D | -0.0207 | -0.0207 |
| *Amblygobius phalaena* | D | -0.0207 | -0.0207 |
| *Anampses caeruleopunctatus* | D | -0.0328 | -0.0269 |
| *Anampses meleagrides* | D | -0.0113 | -0.0097 |
| *Anampses twistii* | D | -0.0162 | 0.0130 |
| *Aphareus furca* | D | -0.0086 | 0.0391 |
| *Apogon angustatus* | N | 0.0000 | 0.0000 |
| *Apolemichthys xanthopunctatus* | D | 0.5360 | 0.3448 |
| *Aprion virescens** | D | 0.4497 | 0.4497 |
| *Arothron meleagris* | C | 0.9977 | 0.9231 |
| *Arothron stellatus* | C | 0.0000 | 0.0000 |
| *Aulostomus chinensis* | C | 0.4497 | 0.4497 |
| *Balistapus undulatus* | D | 0.1499 | 0.1275 |
| *Balistoides viridescens* | D | 0.0000 | 0.0000 |
| *Bodianus axillaris* | D | 0.0597 | 0.1999 |
| *Bodianus loxozonus* | D | -0.0065 | 0.0440 |
| *Bolbometopon muricatum* | D | 0.4497 | 0.4497 |
| *Caesio teres* | D | -0.0278 | -0.0020 |
| *Calotomus carolinus* | D | -0.0025 | 0.0062 |
| *Cantherhines dumerili* | D | 0.0066 | 0.0330 |
| *Canthigaster amboinensis* | D | 0.4497 | 0.4497 |
| *Canthigaster papua* | D | 0.0000 | 0.0000 |
| *Canthigaster solandri* | D | 0.0000 | 0.0000 |
| *Caracanthus maculatus* | D | 0.6608 | 0.7572 |
| *Carangoides orthogrammus* | D | -0.0207 | -0.0207 |
| *Caranx ignobilis** | C | -0.0320 | -0.0344 |
| *Caranx lugubris** | C | -0.0280 | -0.0242 |
| *Caranx melampygus** | D | 0.4010 | 0.1920 |
| *Carcharhinus amblyrhynchos** | C | -0.0479 | -0.0501 |
| *Carcharhinus melanopterus** | C | -0.0525 | -0.0508 |
| *Centropyge flavissima* | D | 0.1841 | 0.2694 |
| *Centropyge loricula* | D | 0.2098 | 0.1864 |
| *Cephalopholis argus* | C | 0.0029 | 0.0019 |
| *Cephalopholis leopardus* | C | 0.4497 | 0.4497 |
| *Cephalopholis miniata* | C | 0.4497 | 0.4497 |
| *Cephalopholis urodeta* | C | -0.0270 | 0.0081 |
| *Chaetodon auriga* | D | -0.0124 | 0.0055 |
| *Chaetodon bennetti* | D | 0.1257 | -0.0052 |
| *Chaetodon ephippium* | D | -0.0319 | -0.0372 |
| *Chaetodon kleinii* | D | 0.0000 | 0.0000 |
| *Chaetodon lineolatus* | C | 0.0000 | 0.0000 |
| *Chaetodon lunula* | C | 0.0970 | -0.0052 |
| *Chaetodon lunulatus* | D | 0.0025 | -0.0229 |
| *Chaetodon meyeri* | D | 0.0094 | -0.0026 |
| *Chaetodon ornatissimus* | D | -0.0085 | -0.0026 |
| *Chaetodon punctatofasciatus* | D | 0.0000 | 0.0000 |
| *Chaetodon quadrimaculatus* | D | -0.0289 | -0.0219 |
| *Chaetodon reticulatus* | D | -0.0436 | -0.0417 |
| *Chaetodon semeion* | D | 0.0000 | 0.0000 |
| *Chaetodon trifascialis* | D | 0.7384 | 1.1184 |
| *Chaetodon ulietensis* | D | 0.1079 | 0.3163 |
| *Chaetodon unimaculatus* | D | 0.0000 | 0.0000 |
| *Chaetodon vagabundus* | D | -0.0207 | -0.0207 |
| *Chanos chanos* | D | -0.0232 | -0.0228 |
| *Cheilinus chlorourus* | D | 0.0000 | 0.0000 |
| *Cheilinus oxycephalus* | D | 0.4497 | 0.4497 |
| *Cheilinus trilobatus* | D | -0.0795 | -0.0637 |
| *Cheilinus undulatus* | D | 0.1301 | 0.3428 |
| *Chlorurus frontalis* | D | -0.0633 | -0.0618 |
| *Chlorurus microrhinos* | D | -0.0183 | -0.0151 |
| *Chlorurus sordidus* | D | -0.0962 | -0.1314 |
| *Chromis acares* | D | -0.0027 | -0.0312 |
| *Chromis margaritifer* | D | 0.0662 | -0.0689 |
| *Chromis vanderbilti* | D | -0.1169 | -0.0931 |
| *Chromis xanthura* | D | -0.0938 | -0.0484 |
| *Chrysiptera glauca* | D | -0.0207 | -0.0207 |
| *Cirrhilabrus exquisitus* | D | -0.0157 | 0.0159 |
| *Cirrhitichthys oxycephalus* | D | 0.0018 | 0.0636 |
| *Cirripectes variolosus* | D | -0.1627 | -0.1172 |
| *Coris aygula* | D | -0.0280 | -0.0290 |
| *Coris centralis* | D | -0.0510 | -0.0537 |
| *Coris gaimard* | D | -0.0927 | -0.0529 |
| *Ctenochaetus binotatus* | D | 0.0000 | 0.0000 |
| *Ctenochaetus cyanocheilus* | D | -0.0528 | -0.0346 |
| *Ctenochaetus hawaiiensis* | D | -0.0280 | -0.0277 |
| *Ctenochaetus marginatus* | D | -0.1026 | -0.0957 |
| *Ctenochaetus striatus* | D | 0.1356 | 0.1533 |
| *Dascyllus auripinnis* | D | 0.0000 | 0.0000 |
| *Dascyllus trimaculatus* | D | 0.0000 | 0.0000 |
| *Elagatis bipinnulata* | D | -0.0207 | -0.0207 |
| *Epibulus insidiator* | D | -0.0629 | -0.0540 |
| *Epinephelus fasciatus* | C | -0.0285 | -0.0166 |
| *Epinephelus howlandi* | C | 0.0000 | 0.0000 |
| *Epinephelus macrospilos* | C | 0.0531 | 0.2157 |
| *Epinephelus malabaricus* | C | 0.0000 | 0.0000 |
| *Epinephelus melanostigma* | C | -0.0151 | 0.1056 |
| *Epinephelus merra* | D | 0.0000 | 0.0000 |
| *Epinephelus polyphekadion** | C | 0.3529 | 0.1534 |
| *Epinephelus spilotoceps* | N | 0.4497 | 0.4497 |
| *Epinephelus tauvina* | C | 0.6190 | 0.6530 |
| *Fistularia commersonii* | D | 0.7949 | 0.8004 |
| *Forcipiger longirostris* | D | 0.1217 | 0.0118 |
| *Gnathodentex aureolineatus* | N | 0.1483 | 0.0093 |
| *Gnatholepis anjerensis* | D | -0.0207 | -0.0207 |
| *Gomphosus varius* | D | -0.0318 | 0.0359 |
| *Gracila albomarginata* | C | -0.0207 | -0.0207 |
| *Gymnothorax flavimarginatus** | C | -0.0207 | -0.0207 |
| *Gymnothorax javanicus* | C | 0.0000 | 0.0000 |
| *Halichoeres hortulanus* | D | -0.0295 | -0.0208 |
| *Halichoeres melasmapomus* | D | 0.0000 | 0.0000 |
| *Halichoeres ornatissimus* | D | -0.0800 | -0.0608 |
| *Helcogramma striata* | D | -0.0423 | -0.0509 |
| *Hemigymnus fasciatus* | D | 0.0173 | 0.0154 |
| *Heniochus acuminatus* | D | 0.0000 | 0.0000 |
| *Hipposcarus longiceps* | D | -0.0202 | -0.0197 |
| *Hologymnosus doliatus* | D | 0.0000 | 0.0000 |
| *Kyphosus cinerascens* | D | -0.0327 | -0.0281 |
| *Labroides bicolor* | D | 0.0244 | 0.2444 |
| *Labroides dimidiatus* | D | -0.1546 | -0.0951 |
| *Labroides pectoralis* | D | 0.0000 | 0.0000 |
| *Labroides rubrolabiatus* | D | 0.0191 | 0.1299 |
| *Labropsis xanthonota* | D | 0.0000 | 0.0000 |
| *Lethrinus xanthochilus* | C | -0.0400 | -0.0330 |
| *Lutjanus bohar** | C | -0.1294 | -0.0862 |
| *Lutjanus fulvus* | C | -0.0092 | -0.0161 |
| *Lutjanus gibbus* | C | -0.0661 | -0.0748 |
| *Lutjanus monostigma* | C | 0.2993 | 0.1165 |
| *Macropharyngodon meleagris* | D | -0.0724 | -0.0566 |
| *Manta birostris* | C | 0.0000 | 0.0000 |
| *Melichthys niger* | D | -0.0380 | -0.0306 |
| *Melichthys vidua* | D | -0.0290 | 0.0046 |
| *Monotaxis grandoculis* | N | 0.1653 | 0.0128 |
| *Mulloidichthys flavolineatus* | N | 0.0584 | 0.0165 |
| *Mulloidichthys mimicus* | D | -0.0207 | -0.0207 |
| *Mulloidichthys vanicolensis* | N | -0.0112 | -0.0111 |
| *Myripristis adusta* | N | 0.7257 | 0.7665 |
| *Myripristis amaena* | N | 1.2029 | 0.9327 |
| *Myripristis berndti* | N | 0.2806 | 0.3475 |
| *Myripristis pralinia* | N | 1.2029 | 0.9327 |
| *Myripristis woodsi* | N | 0.4497 | 0.4497 |
| *Naso brevirostris* | D | -0.0207 | -0.0207 |
| *Naso hexacanthus* | D | -0.0343 | -0.0322 |
| *Naso lituratus* | D | -0.0246 | 0.0024 |
| *Naso unicornis* | D | -0.0289 | -0.0295 |
| *Nemateleotris magnifica* | D | -0.0382 | -0.0382 |
| *Neoniphon opercularis* | N | 0.0915 | 0.0593 |
| *Neoniphon sammara* | N | 0.0832 | 0.1191 |
| *Novaculichthys taeniourus* | D | -0.0280 | -0.0293 |
| *Ostracion meleagris* | D | -0.0207 | -0.0207 |
| *Oxycheilinus unifasciatus* | D | 0.0461 | 0.1457 |
| *Paracirrhites arcatus* | D | -0.0169 | 0.0292 |
| *Paracirrhites forsteri* | D | 0.0525 | 0.0440 |
| *Paracirrhites hemistictus* | D | -0.0339 | 0.0805 |
| *Parupeneus barberinus* | D | -0.0263 | -0.0273 |
| *Parupeneus cyclostomus* | C | -0.0280 | -0.0225 |
| *Parupeneus insularis* | N | -0.0136 | 0.1011 |
| *Parupeneus multifasciatus* | D | -0.0365 | 0.0208 |
| *Pempheris oualensis* | N | 1.0768 | 0.5167 |
| *Pervagor janthinosoma* | D | -0.0207 | -0.0207 |
| *Plagiotremus rhinorhynchos* | D | 0.0000 | 0.0000 |
| *Plagiotremus tapeinosoma* | D | 0.0480 | 0.0732 |
| *Platax boersii* | D | 0.0000 | 0.0000 |
| *Plectroglyphidodon dickii* | D | 0.3807 | 0.4237 |
| *Plectroglyphidodon imparipennis* | D | -0.0207 | -0.0207 |
| *Plectroglyphidodon johnstonianus* | D | -0.0410 | -0.0258 |
| *Pomacanthus imperator* | D | 0.6568 | 0.6282 |
| *Pomacentrus coelestis* | D | -0.0782 | -0.0524 |
| *Priacanthus hamrur* | N | 0.4497 | 0.4497 |
| *Pseudanthias bartlettorum* | D | 0.2283 | 0.1189 |
| *Pseudanthias dispar* | D | -0.0438 | -0.0349 |
| *Pseudanthias olivaceus* | D | -0.0158 | -0.0102 |
| *Pseudobalistes flavimarginatus* | D | 0.0000 | 0.0000 |
| *Pseudocheilinus hexataenia* | D | 0.1402 | -0.0638 |
| *Pseudocheilinus octotaenia* | D | -0.0568 | -0.0010 |
| *Pseudocoris heteroptera* | D | 0.0000 | 0.0000 |
| *Pseudodax mollucanus* | D | 0.9977 | 0.8901 |
| *Ptereleotris evides* | D | -0.0207 | -0.0207 |
| *Ptereleotris heteroptera* | D | 0.0000 | 0.0000 |
| *Pterocaesio lativittata* | D | -0.0280 | -0.0280 |
| *Pterocaesio tile* | D | 0.1260 | 0.0267 |
| *Rhinecanthus aculeatus* | D | 0.4497 | 0.4497 |
| *Sargocentron caudimaculatum* | N | 0.1554 | 0.2228 |
| *Sargocentron microstoma* | N | 0.7606 | 0.7300 |
| *Sargocentron spiniferum* | N | 0.0385 | 0.2829 |
| *Sargocentron tiere* | N | -0.0159 | 0.0852 |
| *Scarus altipinnis* | D | -0.0054 | 0.0052 |
| *Scarus forsteni* | D | -0.0207 | -0.0207 |
| *Scarus frenatus* | D | 0.0310 | 0.0404 |
| *Scarus ghobban* | D | 0.0165 | 0.0220 |
| *Scarus globiceps* | D | -0.0625 | -0.0624 |
| *Scarus niger* | D | -0.0207 | -0.0207 |
| *Scarus oviceps* | D | -0.0742 | -0.0716 |
| *Scarus psittacus* | D | -0.0145 | -0.0435 |
| *Scarus rubroviolaceus* | D | 0.0066 | 0.0626 |
| *Scarus schlegeli* | D | 0.0000 | 0.0000 |
| *Scarus spinus* | D | -0.0262 | -0.0262 |
| *Scarus tricolor* | D | -0.0358 | 0.0182 |
| *Sebastapistes cyanostigma* | C | 0.2717 | 0.0971 |
| *Sphyraena barracuda** | C | -0.0207 | -0.0207 |
| *Stegastes aureus* | D | -0.1490 | -0.1308 |
| *Stegastes fasciolatus* | D | -0.0295 | -0.0295 |
| *Stegastes nigricans* | D | 0.0000 | 0.0000 |
| *Stethojulis bandanensis* | D | -0.0709 | -0.0417 |
| *Stethojulis interrupta* | D | -0.0269 | -0.0264 |
| *Sufflamen bursa* | D | 0.0066 | -0.0062 |
| *Sufflamen chrysopterum* | D | -0.0622 | -0.0620 |
| *Synodus variegatus* | C | -0.0295 | -0.0248 |
| *Taeniura meyeni* | D | 0.0000 | 0.0000 |
| *Thalassoma amblycephalum* | D | -0.0506 | -0.0580 |
| *Thalassoma hardwicke* | D | 0.0134 | -0.0204 |
| *Thalassoma lunare* | D | -0.0295 | -0.0215 |
| *Thalassoma lutescens* | D | -0.0636 | 0.0267 |
| *Thalassoma quinquevittatum* | D | -0.1797 | -0.1117 |
| *Triaenodon obesus** | C | -0.0207 | -0.0207 |
| *Valenciennea strigata* | D | -0.0295 | -0.0281 |
| *Variola louti** | C | 0.0552 | 0.0135 |
| *Xanthichthys caeruleolineatus* | D | 0.0000 | 0.0000 |
| *Zanclus cornutus* | D | 0.0484 | 0.0336 |
| *Zebrasoma rostratum* | D | 0.0742 | 0.1538 |
| *Zebrasoma scopas* | D | 0.0489 | -0.0161 |
| *Zebrasoma velifer* | D | 0.0314 | 0.0047 |

LITERATURE CITED IN SUPPORTING INFORMATION

Compagno LJV (1984) FAO Species Catalogue. Sharks of the world. Vol. 4. Carcharhiniformes. An annotated and illustrated catalogue of shark species known to date. FAO Fisheries Synopsis 125:251-655

Gillibrand CJ, Harris AR, Mara E (2007) Inventory and spatial assemblage study of reef fish in the area of Andavadoaka, South-West Madagascar (Western Indian Ocean). Western Indian Ocean J Mar Sci 6:183-197

Harmelin-Vivien ML, Bouchon C (1976) Feeding behaviour of some carnivorous fishes (Serranidae and Scorpaenidae) from Tulear (Madagascar). Mar Biol 37:329-340

Heemstra PC, Randall JE (1993) FAO Species catalogue. Vol. 16. Groupers of the world (family Serranidae, subfamily Epinephelinae). An annotated and illustrated catalogue of the grouper, rockcod, hind, coral grouper and lyretail species known to date. FAO Fisheries Synopsis 125(16)

Hiatt RW, Strasburg DW (1960) Ecological relationships of the fish fauna on coral reefs of the Marshall Islands. Ecol Monographs 30:65-127

Hobson ES (1974) Feeding relationships of teleostean fishes on coral reefs in Kona, Hawaii. Fish Bull 72:915-1031

Holland KN, Lowe CG, Wetherbee BM (1996) Movements and dispersal patterns of blue trevally (Caranx melampygus) in a fisheries conservation zone. Fish Res 25:279-292

Kuiter RH, Tonozuka T (2001) Pictorial guide to Indonesian reef fishes. Part 2. Fusiliers - Dragonets, Caesionidae – Callionymidae. Zoonetics, Australia

Lieske E, Myers R (1994) Collins Pocket Guide. Coral reef fishes. Indo-Pacific & Caribbean including the Red Sea. HarperCollins Publishers

Marnane MJ, DR Bellwood (2002) Diet and nocturnal foraging in cardinalfishes (Apogonidae) at One Tree Reef, Great Barrier Reef, Australia. Mar Ecol Prog Ser 231:261-268

Myers R (1999) Micronesian reef fishes. Coral Graphics, Barrigada, Guam

Randall J (2005) Reef and shore fishes of the South Pacific. University of Hawaii Press, HI

Randall JE, Allen GR, Steene RC (1990) Fishes of the Great Barrier Reef and Coral Sea. University of Hawaii Press, Honolulu, Hawaii

Shpigel M, Fishelson L (1989) Food habits and prey selection of three species of groupers from the genus Cephalopholis (Serranidae: Teleostei). Environ Biol Fishes 24:67-73

Steene RC (1978) Butterfly and angelfishes of the world. A. H. and A. W. Reed Pty Ltd., Sydney, Australia
