# Supplementary material for: Night Shift: Expansion of Temporal Niche Use Following Reductions in Predator Density
Source: PLoS One. 2012 Jun 13;7(6):e38871. doi: 10.1371/journal.pone.0038871 (PMC3374761; doi:10.1371/journal.pone.0038871)
Supplement: Text S1 — Mechanics of Cohen's D effect size measurements used to compare the density and biomass of fish species on the reefs of Palmyra and Tabuaeran. (DOCX) [file pone.0038871.s002.docx]

SUPPORTING INFORMATION

**Supporting Text S1.** The density and biomass of diurnal, cathemeral, and nocturnal fish species were compared between Palmyra and Tabuaeran using Cohen’s D effect size measurements as:


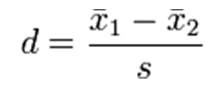


Where:


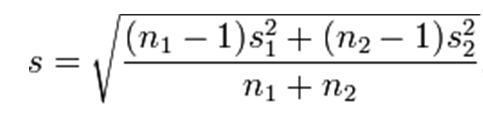


And:


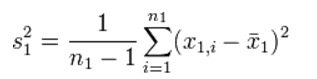


Values from Tabuaeran were set as χ_1_ and Palmyra as χ_2_ such that positive effect size values represent increases in fish density or biomass at Tabuaeran.
